# Does Disinformation Toward Women Politicians Reflect Gender Stereotypes? Exploring the Role of Leaders’ Political Orientations

**DOI:** 10.3390/bs15050695

**Published:** 2025-05-17

**Authors:** Carmela Sportelli, Francesca D’Errico

**Affiliations:** 1 Faculty of Psychology, Uninettuno Telematic International University, 00186 Rome, Italy; 2 For.Psi.Com, University of Bari “Aldo Moro”, 70121 Bari, Italy; francesca.derrico@uniba.it

**Keywords:** sexism, political gender gap, information disorders, sexist stereotypes, disinformation

## Abstract

The recent literature highlights the importance of implementing ad hoc media literacy initiatives to counter sexist stereotypical representations in social media, particularly within the political field. To this end, the present study focuses on false and misleading contents (information disorders) about female politicians, since they can reflect gender stereotypes, portraying women as unfit for political roles. Thus, our exploratory study aims to analyze the stereotype content of information disorders targeting Italian female politicians, following classic models of stereotype content. Furthermore, it seeks to explore the role played by the type of information disorder and the political orientation of the targeted leaders. A database of 120 information disorders have been collected, codified, and analyzed. The results highlight that information disorders predominantly target the dimension of communion. Focusing on the types of information disorders, fabricated and misleading content appears to be associated with the professional and private life domains, primarily conveying communion-based stereotypical representations of politicians. Satirical and parodic contents, on the other hand, were associated with the esthetic domain, conveying agency-based stereotypical representations, with a particular focus on politicians’ physical competence. Regarding political orientation, a “Stereotyping from my eyes” effect was observed: communion-based stereotypical content prevails in information disorders targeting conservative leaders, while progressive leaders are stereotyped concerning the agency dimension. This effect may reveal a difference between conservative and progressive audiences in their adherence to traditional gender roles.

## 1. Introduction

Promoting inclusive and healthy digital environments is a fundamental challenge in today’s world. To this end, the implementation of strategies to foster media literacy and prevent the dissemination of distorted and stereotypical representations in the media, particularly on social media, is becoming increasingly crucial. In this regard, the study of the modalities and characteristics that this type of content assumes in digital environments plays a critical role, as such insights can inform preventive psychological interventions (D’Errico et al., 2024). According to the literature, fake news and information disorder, defined as misleading, false or harmful news (Wardle & Derakhshan, 2017), have received considerable attention in research, especially with the growing use of new media and the associated concerns regarding their detrimental impact on democracy. Research has demonstrated that information disorders spread due to new technologies, which amplify the natural polarization of conversations within echo chambers (Sunstein, 2001) and filter bubbles (Pariser, 2011). Moreover, fake news and information disorders often mimic genuine news in both content (Riva, 2024) and form (Lazer et al., 2018), exploiting stereotypes, cognitive shortcuts, and bias.

A further essential aspect of information disorders lies in their implications for the spread of stereotypes and prejudices, as is also evident in other research areas such as racial disinformation. For example, the literature highlights that the sharing of ‘racial hoaxes’ promotes the dissemination of stereotypes about immigrants, associating them with the lack of benevolence (perceived social dangerousness), a lack of competence or power that threatens the national economy, and even ‘physical’ threat, portraying immigrants as “plague-spreaders” (D’Errico et al., 2022). Consistent with these findings, C. L. Wright and Duong (2021) pointed out that exposure to racial fake news correlates with a reinforcement of ethnic bias.

Regarding gender, however, analyses of traditional communication channels such as TV and advertisement (Haraldsson & Wängnerud, 2019; Santoniccolo et al., 2023; Tartaglia & Rollero, 2015; Volpato, 2022) reveal a significant penalization of women, especially when they aspire to positions of power that fall outside traditional gender roles, such as political roles. Specifically, prejudices (Heldman et al., 2018) and gender stereotypes manifest in the quantity and manner in which the media treat female political figures, even to the point of applying specific forms of delegitimization portraying them as insufficiently or excessively masculine (Heldman et al., 2018) and less competent than their male counterparts (Valentino et al., 2018).

However, relatively little research has investigated the relationship between gender stereotypes and information disorders, specifically how the latter contribute to reinforcing sexist stereotypes and, consequently, which strategies might be the most effective in countering these stereotypical representations.

Initial evidence shows that a greater amount of fake news is shared concerning female politicians, compared to their male counterparts (Allcott & Gentzkow, 2017; Stabile et al., 2019). This suggests that the representation of female politicians in information disorders may mirror patterns found in the mainstream media, conveying “double bind” representations (Stabile et al., 2019).

Building upon these studies, the aim of this research is to investigate the relationship between different types of information disorders and the dimensions of stereotyping that target women political figures in the Italian context, with a specific focus on the potential role of the leaders’ political orientation. In particular, we hypothesize that the stereotypical attack will align with the ideological beliefs and leadership traits prioritized by the potential audience, whose political orientation is presumed to be opposite to the targeted leader’s ones (an effect we will refer to as “Stereotyping from my eyes” from now on). Political orientation of both voters and politicians could play an essential role in this process, since previous research has shown that conservative and progressive audience adhere to traditional values and gender-based expectations on female candidates differently (Carpinella & Johnson, 2013; Jost et al., 2003, 2018; Lye & Waldron, 1997).

To achieve this goal, the definitions of information disorder (Wardle, 2018) will be applied to detect the level of manipulation within an Italian database. Furthermore, stereotype contents will be codified, integrating the two main dimensions emerging from the Stereotypes Content Model and Dual Perspective Model (Abele & Wojciszke, 2014; Fiske et al., 2007), following their application in the domain of social media ethnic stereotypes (Bosco et al., 2023), and adapting them to the domain of gender stereotypes.

Thus, this study aims to deepen the role played by media biases and sexist stereotypes associated with information disorder as a first step to counter gendered disinformation, in line with previous studies focused on ethnic stereotypes detection in the context of racial disinformation prebunking (see for instance, D’Errico et al., 2024).

### 1.1. Gender Gap and Sexist Stereotypes in Political Media Coverage

The media plays a key role in disseminating sexist and stereotyping representations (Haraldsson & Wängnerud, 2019; Santoniccolo et al., 2023; Tartaglia & Rollero, 2015). The literature highlights that stereotype-consistent news about social groups can negatively impact attitudes towards those groups. For instance, Arendt (2023) found that forced exposure to stereotype-consistent news about Afghan asylum seekers increased prejudice, whereas forced exposure to counter-stereotypical content reduced it. Similarly, Saleem et al. (2017) found a positive correlation between exposure to stereotypical representations of Muslims in news and support for military interventions in Muslim-majority countries, as well as for domestic and international policies detrimental to Muslims. In particular, the media influences women’s social perception by selecting and emphasizing certain information; for example, by underrepresenting women in leadership roles and conveying stereotypical and sexist representations, and using unequal language (Volpato, 2022). Gender inequality in media representation has been shown to hinder political (Haraldsson & Wängnerud, 2019) and career (Steinke, 2017) ambition. Furthermore, heavy media consumption has been associated with a stronger endorsement of traditional gender roles and norms (Scharrer & Warren, 2022). In the online context as well, sexism and gender stereotypes are conveyed in various forms, such as sexist hate speech and memes (Lingiardi et al., 2020; Siddiqi et al., 2018), eliciting reactions linked to moral disengagement and other condemning emotions (Paciello et al., 2021; Poggi & D’Errico, 2018).

Within this context, gender stereotypes can be conceptualized using classic models of stereotype content: Stereotypes Content Model and Dual Perspective Model of Agency and Communion (Abele & Wojciszke, 2014; Fiske et al., 2007). These models identify two primary dimensions that describe stereotype content: Agency/Competence and Communion/Warmth. According to this theoretical framework, agency relates to intellectual activities, personal autonomy, skills, efficiency in goal attainment, and assertiveness, while communion relates to social activities, loyalty, cooperation, morality, and nurturance. Stereotypically, men are believed to possess agentic traits, like being dominant, assertive, and competent, while women are associated with high levels of communal qualities, like being friendly, warm, and nurturant, and low levels of agentic ones, being described as passive, emotional, and illogical (Eagly & Karau, 2002; Ellemers, 2018). In the political field, sexist and stereotypical representations can be particularly detrimental: political leadership is usually considered a masculine role, requiring agentic traits (Koenig et al., 2011). According to the Role Congruity Theory, the perceived mismatch between the female stereotyped traits (communal) and the features usually associated with political leadership (agentic) lead to an evaluation of women as less suitable for political roles (Eagly & Karau, 2002). Thus, women leaders are caught in a double bind (Jamieson, 1995), inevitably failing to meet stndards, because they either violate the stereotype of a leader or that of a woman (Van der Pas & Aaldering, 2020). In fact, if women demonstrate themselves as effective leaders, they are considered to be violating gender standards by exhibiting male-stereotypical, agentic traits while failing to manifest female-stereotypical, communal characteristics. This can lead to an unfavorable evaluation for gender role violation, especially by those who endorse traditional gender roles (Eagly & Karau, 2002). This reaction reflects the general tendency for violations of injunctive norms to generate disapproval (Cialdini & Trost, 1998). On the other hand, conforming to communal expectations can undermine their leadership success. According to the literature (Eagly & Karau, 2002), prejudice against female leaders and potential leaders manifests in two ways: (a) a negative evaluation of women’s (compared to men’s) leadership potential because leadership skills are more stereotypically associated with men, and (b) a negative evaluation of women’s actual leadership behavior because such behavior is perceived as less desirable in women than men. Women leaders’ options are thus limited by threats from two directions: either they fail to satisfy the requirements of their leader role if they conform to their gender role (showing communal traits), or they fail to meet the requirements of their gender role if they conform to their leadership role (showing agentic traits). Both of these situations would result in less positive perceptions of female leaders and potential leaders compared to men.

Consequently, women in political roles are forced to find an acceptable balance between masculine and feminine traits in order to perform their role ‘acceptably’. Otherwise, if they disconfirm stereotypical expectations, they are undervalued as a form of backlash (Ellemers, 2018; Rudman & Glick, 1999; K. A. M. Wright & Holland, 2014).

Media coverage of men and women in the political field appears to align with these stereotypical representations. For example, Van der Pas and Aaldering (2020) found that female politicians receive more appearance and family coverage than their male counterparts, their gender is more frequently mentioned, and their combative behavior is evaluated as exaggerated. This sexist media coverage clearly harms women in politics, causing them to lose support and be perceived as less successful, and any mention of a female candidate’s appearance in the media has been demonstrated to be detrimental to the politician’s perception (Bates, 2015). In fact, contents that focus on a politician’s personality, attractiveness, or family and home life alienate the candidate from political topics—perhaps even from political relevance. The media’s emphasis on female candidates’ personality and appearance diverts attention away from political issues, leading to a negative perception of the candidates’ credibility and competence (Dunaway et al., 2013; Lawrence, 2000; Woodall & Fridkin, 2007). On the other hand, when female politicians’ competence and leadership skills are in question, they risk being perceived too agentic and power-seeking, eliciting moral outrage from voters (Heldman et al., 2018). In this context, Conroy et al. (2015) suggest that the increasing use of new media, as well as more online coverage and campaigning, could worsen the situation for female candidates, since the lack of editorial filters and ad hoc fact-checking procedures could lead to more gendered and sexist coverage.

The political orientation of both voters and politicians could play an essential role in the previously described negative impact of gender-based expectations on female candidates. Previous research has shown that conservatism is characterized by adherence to traditional values: Lye and Waldron (1997) found that conservative beliefs are associated with more traditional attitudes toward gender roles, which aligns with the conservative tendency to view society as hierarchically organized, with certain groups ranked lower than others based on their ascribed characteristics (Jost et al., 2003, 2018). Focusing on gendered political perception, ideological differences between conservatives and liberals can have an impact: for example, conservative women competing for the United States House of Representatives appeared more sex-typical (i.e., stereotypically feminine) than their liberal counterparts (Carpinella & Johnson, 2013). Furthermore, sex-typicality has been shown to predict political success for female politicians, particularly in states with an extensive conservative electorate (Hehman et al., 2014).

### 1.2. Information Disorders and Their Stereotypes in the Political Field

The increasing use of new media has amplified the circulation of misleading information, also during political campaigns (Hobbs & McGee, 2014), thereby inferring with democratic institutions and processes (Paul & Matthews, 2016). Wardle and Derakhshan (2017) defined information disorder as the amount of misleading, false, or harmful news spread through social media. The concept of information disorder describes a fluid spectrum of content, including satiric, parodic, misleading, and fabricated items (Wardle, 2018). Considering the broader spectrum of information disorders allows us to move beyond the narrow definition of “fake news”, thus encompassing different forms of problematic and dangerous content online, including inaccurate content that can be misleading (including factually correct information presented in a distorted manner), manipulated, or entirely fabricated (Wardle, 2018).

In the political field, information disorders can influence public opinion and voting behaviors (Allcott & Gentzkow, 2017; Pennycook & Rand, 2019). For example, Zimmermann and Kohring (2020) found that, during the 2017 German parliamentary election, people who believed in disinformation were more likely to vote for right-wing populists over the main governing party. Furthermore, information disorders can be used to amplify political polarization (Petla, 2023), even inciting violence and civil unrest (Pérez-Escolar & Noguera-Vivo, 2021). Sharing political content that qualifies as information disorder can be used to signal one’s political affiliation by attacking political opponents or mobilizing politically aligned peers. In this context, information disorders can be particularly useful because this type of content is not limited by reality or objectivity, allowing it to be excessively harsh in its portrayal of political opponents (Acerbi, 2019; Osmundsen et al., 2021).

As previously pointed out, media content can convey stereotypes, which also applies to information disorders. D’Errico et al. (2022) highlighted how misleading racial news, defined as “racial hoaxes”, generally convey linguistic forms of stereotypes and prejudices aimed at dehumanizing and attributing various types of threats to their protagonists. Furthermore, C. L. Wright and Duong (2021) found that exposure to racial information disorders can reinforce anti-immigrant attitudes. Focusing on gender stereotypes, the body of research on this topic is still limited, exemplified by Herrero-Diz et al. (2020), which investigates common features of Spanish information disorders targeting ordinary women, finding how this type of content mainly aims to undermine the credibility and dignity of feminist claims, while questioning the severity of sexist behaviors, including gender-based violence. Sexist information disorders can be particularly dangerous since they can reinforce messages that incite hatred (hate speech) against women. Gendered disinformation and sexist hate speech seem to be interconnected and mutually reinforcing within online discourse. Disinformation can provide a false basis for hateful rhetoric, while hate speech can be embedded within misinformative narratives to delegitimize and attack feminist perspectives and women in general (Raquel et al., 2024). For example, Raquel et al. (2024) highlighted how sexist hate speech and gendered disinformation are used within social media to undermine the feminist movement by portraying it as irrational or aggressive.

In the political field, to the best of our knowledge, only Stabile et al. (2019) have explored the potential role of information disorders in conveying sexist stereotypes. In particular, they focused on fake news targeting Hillary Clinton, analyzing the specific case of the 2016 U.S. election campaign, highlighting how these contents are connected to the playbook of sexist and stereotyping portrayals of female candidates in traditional media. Specifically, the authors analyzed two case studies of fake news stories targeting Clinton, revealing representations consistent with the double bind that affects female politicians, portraying Clinton as physically weak or, conversely, supporting the “Lady Macbeth narrative”, that villainizes power-seeking women by portraying them as immoral and outrageous.

Thus, this limited literature suggests that information disorders can convey sexist and stereotyping representations of female politicians, potentially causing a serious blurring in the evaluation of their political and institutional competence. Nevertheless, to the best of our knowledge, these aspects have never been investigated in terms of sexist stereotype contents, deepening the role played by women leaders’ political orientation.

## 2. Materials and Methods

### 2.1. Overview and Objectives

The present study aims to explore the characteristics of information disorders targeting female politicians, since the literature shows in other stereotype domains (C. L. Wright & Duong, 2021) that information disorders can reinforce stereotypical narratives; thus, we hypothesize that information disorders targeting female politicians may reinforce sexist stereotypes, aligning with studies on the political gender gap, in this case referring to the Italian political context.

In particular, the study is based on the collection of a sample of different informative disorders, since, to the best of our knowledge, the majority of the studies are based on case studies focusing on particular political events (Stabile et al., 2019), neglecting aspects associated with the stereotype content and a variety of characteristics related to the cultural background and the political orientation of the women leaders.

In order to deepen the thematic and linguistic features of information disorders, we first present a preliminary analysis on the lexicon emerging from the collected contents. Furthermore, we focus on investigating the features of information disorders targeting Italian female politicians, considering stereotype content dimensions and sub-dimensions (Abele & Wojciszke, 2014; Bosco et al., 2023; Fiske et al., 2007) (RQ), focusing on (a) the role played by women leaders’ political orientation in shaping sexist stereotypical content of information disorders (RQ1); (b) the role played by the information disorders’ type (RQ2).

### 2.2. Collecting Procedure and Coding

To investigate these objectives, we collected 120 information disorders targeting Italian female politicians from debunking websites (“Bufale.net”, “Facta”, “Open”, “Butac”), Google Fact Check Explorer (a Google tool that collects news debunked) and other sources. The information disorders refer to a time span ranging from May 2014 to June 2024. Specifically, searches were conducted using names associated with important Italian female politicians, beginning with the two most significant figures in the current political landscape, “Giorgia Meloni” and “Elly Schlein” (others names used include: “Lucia Azzolina”, “Laura Boldrini”, “Maria Elena Boschi”, “Ilaria Capua”, “Monica Cirinnà”, “Elsa Fornero”, “Ilaria Cucchi”, “Ilaria Salis”, “Luciana Lamorgese”, “Daniela Santanchè”, “Liliana Segre”).

The inclusion and exclusion criteria were as follows:

- Inclusion criteria: the content falls under the definition of information disorder and is about Italian female politicians.
- Exclusion criteria: the content does not meet the definition of information disorder or involves an Italian men politician, a non-political Italian woman, or non-Italian men/female politicians.

All information disorders were collected by extracting information such as publication data (where available), the targeted politician and her political orientation (progressive vs. conservative), the main topic of the news (professional competence vs. private life vs. esthetic) and a link to the article. The coding procedure started with creating a grid for the relevant variables (type of information disorder; dimensions and sub-dimensions of the stereotype content) based on the relevant theoretical models. Thereafter, two expert independent judges (a PhD student in social psychology and a communication expert) coded information disorders for topic of the news, type of information disorder, and stereotype content (dimensions and sub-dimensions). All judges discussed the coding criteria starting from the subset of 60 information disorders and established a common coding grid. Subsequently, they separately encoded the corpus basing on the grid. The inter-judges agreements could be considered “strong” for the topic of the news (κ Cohen = 0.82) and “good” for type of information disorder (κ Cohen = 0.75), and stereotype content (κ Cohen = 0.73).

#### 2.2.1. Type of Information Disorder

Wardle (2018) classified information disorders into seven categories, forming a spectrum with increasing levels of manipulation. Satire, the least problematic type of information disorder, is on one end of the spectrum, whereas fabricated content is on the other. Thus, in the present study, information disorders were coded as follows:

1. Satire and Parody: satiric or parodic content that people could consider real news.
2. False Connection: contents in which headlines, visuals or captions do not support the article’s information (like clickbait headlines).
3. Misleading Content: contents in which accurate information is framed to inaccurately represent an issue or an individual (e.g., “SCHLEIN RIDES ON THE GAY PRIDE FLOAT: IT’S THE BIBBIANO’S PARTY—VIDEO”).
4. False Context: accurate contents that circulated out of their original context, misleading the reader.
5. Imposter Content: contents that improperly used organizations’ logos or journalists’ bylines.
6. Manipulated Content: genuine contents that are manipulated to deceive (e.g., two genuine images spliced together to manipulate the reader or convey a certain message).
7. Fabricated Content: false contents meant to propagate incorrect information (e.g., “She wants to take money from the poor and workers but earns €25,000 a month. Meloni cuts YOUR income!”).

#### 2.2.2. Stereotype Content

**Figure 1:**
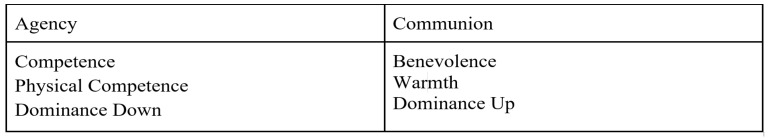
Stereotype sub-dimensions distribution in the Agency and Communion dimensions.

To analyze the stereotypical portraits of female politicians, we adopted the two main dimensions emerging from the Stereotype Content Model and Dual Perspective Model (Abele & Wojciszke, 2014; Fiske et al., 2007), henceforth Agency and Communion. Following the work of Bosco et al. (2023), we coded the agency and communion sub-dimensions, integrating the aforementioned theoretical models with the two Dominance’s related discredit mechanisms (up and down) in Poggi et al. (2011). They were, as illustrated in Figure 1, distinguished as follows: the communion dimension includes benevolence, warmth and dominance up sub-dimensions, while the agency dimension includes competence, physical competence, and dominance down (Abele et al., 2016; Bosco et al., 2023; Signorello et al., 2012).

Thus, each stereotype sub-dimension was codified as being related to either Agency or Communion (Figure 1), allowing us to consider two main stereotypical representations of female politicians. The first is characterized by a lack of agency, which is conveyed by discrediting the target based on her cognitive and physical competence and describing her as irrelevant and useless; the second is characterized by a lack of communion, which is conveyed by portraying the target as immoral, dishonest, emotionally distant, or excessively dominant (to the point of being considered aggressive and overbearing).

Following this classification, the stereotype sub-dimensions were coded as follows:

1. Benevolence: the target is portrayed as dishonest, immoral, illegal, unethical (e.g., “Elly Schlein’s PD stands with thieves: what this photo reveals”. Note: “PD” stands for “Democratic Party”.).
2. Warmth: the target is portrayed as emotionally distant, callous, heartless.
3. Competence: the target is portrayed as stupid, incompetent.
4. Physical Competence: the focus is on the target’s appearance and how it deviates from esthetic standards.
5. Dominance Up: the target is portrayed as overly dominant, arrogant, overbearing (e.g., “The school will be open until the final teacher is alive.” Citation attributed to politician Lucia Azzolina).
6. Dominance Down: the target is portrayed as nondominant, irrelevant, useless (e.g., “European elections, the PD hides Schlein’s name from the party symbol. They are ashamed of her.”).
7. No stereotyping: the content has no stereotyping purposes.

## 3. Results

### 3.1. Preliminary Qualitative Lexicon Analysis

A preliminary qualitative analysis of the corpus’ lexicon highlights the frequent use of colloquial and vulgar vocabulary. Some examples of words used are “jerks, needing a tso, gobbledygook, asshole”. These words recur in concordances like

“Boldrini asked Draghi to provide migrants with a 500 euros dignity income each month. **This lady needs a TSO**”.

One of the main topics of information disorders is related to immigration, Islam and foreign affairs, using words like “weapons, illegal migrants, war, Ukraine, Islamic, Islamization, Africa, hijab, immigrants”. Furthermore, one of the most common stereotype dimensions in this corpus is closely linked to the dimensions prevalent in the ‘man-osphere’, a virtual galaxy of spaces in which the supremacy of masculinity and the subordination of women are exalted (Van Valkenburgh, 2021), from which emerge the evaluative canons of misogynistic groups (such as the so-called incels) corresponding to the LSM Laws: Look, Status, Money. Thus, the focus on female politicians’ looks is expressed through words related to leaders’ outfits or esthetic features that recur in concordances like “Daniela Santanchè uses a **rejuvenation filter** to impress Draghi”.

The status dimension is expressed by stressing the excessive dominance (Bosco et al., 2023), or the lack of dominance (D’Errico, 2019), of women leaders. They are described as passive, useless, and influenced by others, using words like “masters, to obey, butler”, or as excessively dominant and overbearing, using words like “to beat, to reeducate, fascists, nazis, to force, to prevent”. Examples of concordances related to Status dimension are

“Meloni **obeys her American masters**: Salvini’s concerns about giving weapons to Kyiv”.

“Meloni **butler**: organizes a meeting between Biden and Zelenski during the G7 in Italy”.

“Cirinnà: ‘We will re-educate your children’”.

The third dimension concerning money is expressed through words like “money, income, annuity, to earn, salary”. They are associated with concordances like

“She wants to take money from the poor and workers, and she **earns €25,000 every month**. Meloni cuts your income”.

Finally, the corpus includes a common theme represented by terms connected with emotive, sexual, and familiar dimensions: “heart, to hug, family, son, brother, gay, affective, friends, love, children, girlfriend, lesbian, lgbqt, to marry, sexual”.

Examples of concordances related to these dimensions are

“What if he was the one who brought love back into the Premier’s broken heart?”

“Schlein the lesbian rejoices”.

### 3.2. Corpus Descriptives

**Figure 2:**
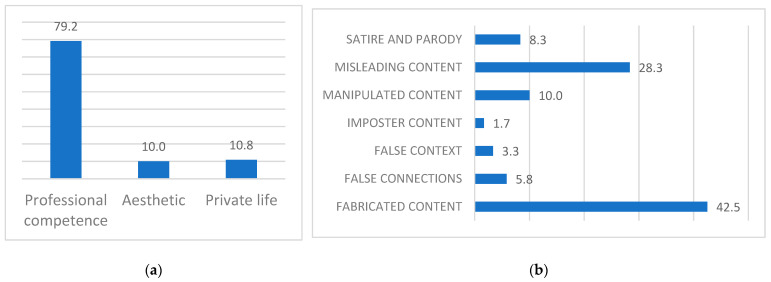
Corpus descriptives: (**a**) Percentages of type of news topic in the collected data. (**b**) Percentages of information disorders in the collected data.

Information disorders target a wide range of Italian female politicians, 57.5% of whom are identified as progressive (e.g., Elly Schlein, Laura Boldrini), while 42.5% are identified as conservatives (e.g., Giorgia Meloni, Daniela Santanchè). The majority of the information disorders is related to the leader’s professional competence (79,2%; n = 95), followed by private life (10.8%, n = 13) and esthetic (10%; n = 12; Figure 2a).The prevalent type of information disorder is fabricated content (42.5%; n = 51), followed by misleading content (28.3%; n = 34); satire and parody contents are also quite commons, representing the 8.3% (n = 10) of the corpus (Figure 2b).

**Figure 3:**
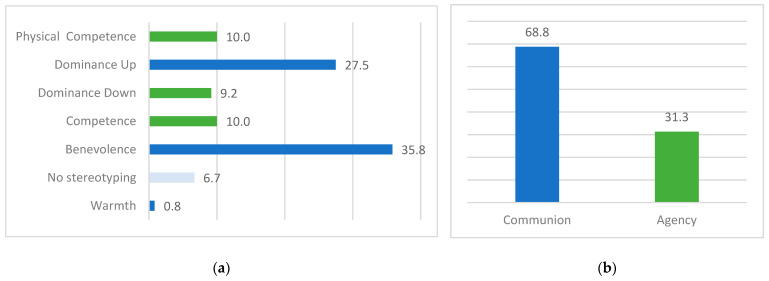
Corpus descriptives: (**a**) Percentages of type of stereotypical sub-dimensions in the collected data. (**b**) Percentages of stereotypical content in the collected data.

In terms of stereotypical sub-dimensions, the most common is Benevolence (35.8%; n = 43), describing politicians as engaging in illegal activities, misleading citizens, disregarding Italian traditions in favor of someone else (usually foreign), or earning exaggerate incomes while ignoring people financial needs. The Dominance Up sub-dimension is also frequent (27.5%; n = 33), followed by Competence (10%; n = 12) and Physical Competence (10%; n = 12), which together represent 20% of the corpus (Figure 3a). Furthermore, the 6.7% (n = 8) of information disorders seems to have no stereotyping aim, focusing on gossips about politicians’ private lives, conveying false and misleading information to persuade social users to believe in phishing contents, or, in a few cases, spreading false and misleading information to provide a positive portrait of the politician (e.g., giving false information excessively positive about the politician’s approval ratings during elections). Considering only stereotyping information disorders (excluding no-stereotyping contents), communion stereotypical representation accounts for 68.8% (n = 77) of the corpus (Agency: 31.3%; n = 35; Figure 3b).

### 3.3. Political Orientation

Regarding news topics, results show how information disorders targeting conservative leaders are mainly focused on the professional area (86.3%) and to a very less extent, on the esthetic and private life (3.9% and 4.2%), while for the progressive leaders contents are focused on professional area are 73.9%, followed by esthetic (14.5%) and private life (6.5%; chi-square test *p* = 0.14; n.s.).

Political orientation and type of information disorders are not significantly associated, since the performed chi square test is not significant (*p* = 0.16): from a descriptive point of view it emerges how in the case of conservative politicians there is a prevalence of fabricated content (39.5%), while in the case of progressive politicians emerges a high level of fabricated (40.6%) and misleading (33.3%) content, and a higher level of satire compared to conservative (11.6% vs. 4.7%).

**Figure 4:**
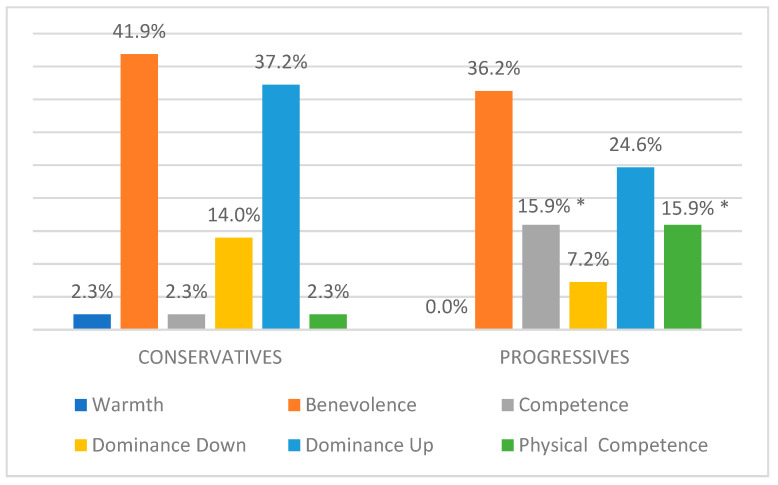
Percentage distribution of stereotypical content × politicians’ political orientation. * significant residuals at |2|.

To investigate the relationship between target leader’s political orientation and stereotypical sub-dimensions expressed through information disorders, a chi-square test [χ^2^ (5) = 13.63; *p* < 0.05] and a maximum likelihood ratio chi-square test [χ^2^ (5) = 16.06; *p* < 0.01] have been performed, considering only stereotyping contents. Both tests reveal a significant association between the target leader’s political orientation and the stereotype content used against her. In particular, even though the Benevolence and Dominance Up stereotype sub-dimensions appear to be prevalent mechanisms in both progressives (36.2% and 24.6%) and conservatives (41.9% and 37.2%), progressive female politicians appear to be more stereotyped in terms of Competence (15.9%) and Physical Competence (15.9%; see Figure 4).

**Figure 5:**
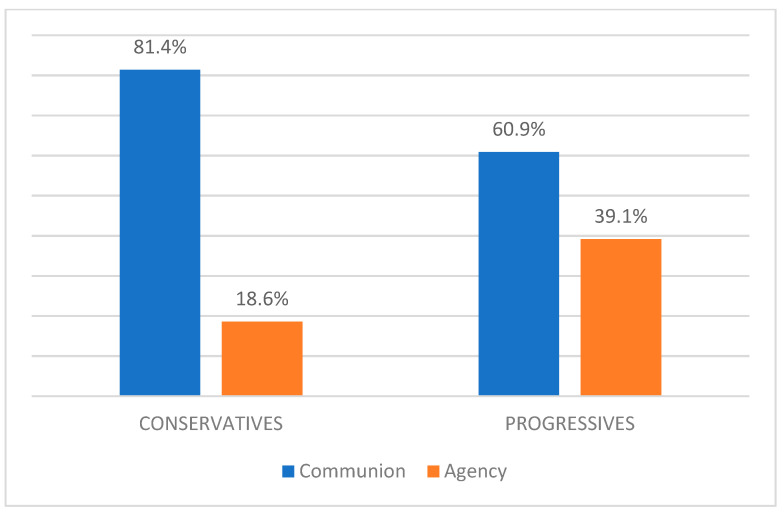
Percentage distribution of stereotypical content in progressive and conservative politicians.

A chi square test [χ^2^ (1) = 5.20; *p* < 0.05] revealed a significant association between target leader’s political orientation and the macro dimensions of stereotype content conveyed by the information disorders. In particular, even though Communion stereotypical representation is prevalent both in conservatives (81.4%) and progressives (60.9%), Agency stereotypical content appears to be prevalent in progressive rather than conservative female politicians (Figure 5).

### 3.4. Information Disorders

For this analysis, different types of information disorder were classified into four categories: “Fabricated content”, “Misleading content”, “Satire and parody”, and “Other” (which included the remaining types of information disorder).

**Figure 6:**
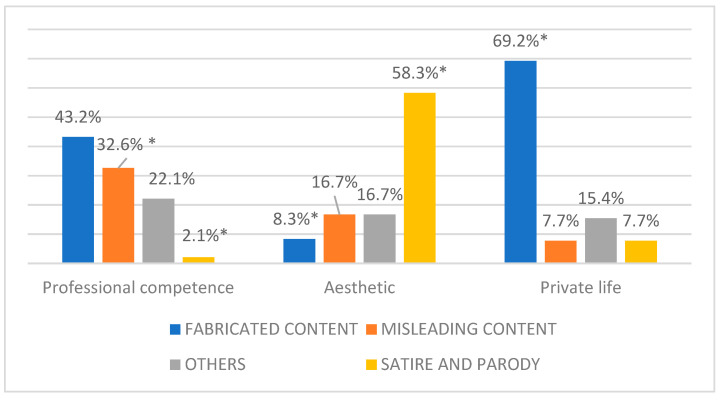
Percentage distribution of information disorders across different news topics. * significant residuals at |2|.

In relation to the type of information disorders, we found a significant association with news topic [χ^2^ (6) = 49.43; *p* < 0.000]. In professional areas, fabricated content and misleading news were the most prevalent (respectively, 43.2% and 32.6%), while, in private life, fabricated content was the most prevalent (69.2%), and satire is strongly associated with the esthetic topics (58.3%; see Figure 6).

**Figure 7:**
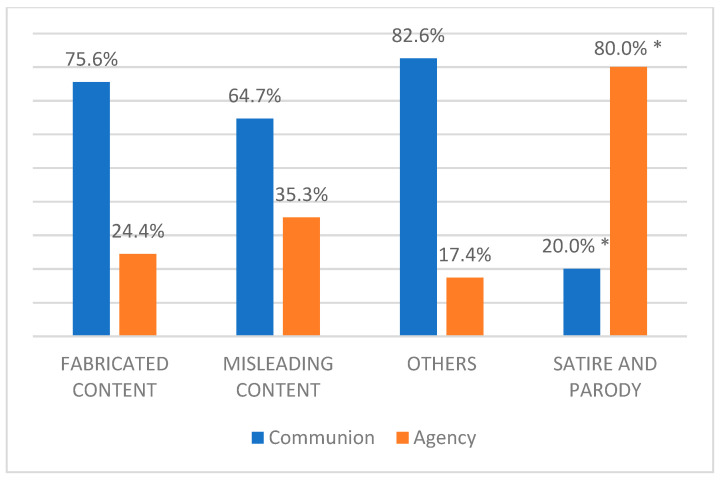
Percentage distribution of stereotypical representations across different types of information disorder. * significant residuals at |2|.

Furthermore, a chi square test [χ^2^ (3) = 14.35; *p* < 0.01] has been performed, revealing a significant association between types of information disorder and stereotypical representations of female politicians. Results highlights that, while fabricated content, misleading content, and other types of information disorder convey mostly communion stereotypical representation (respectively, communion represents 75.6%, 64.7%, 82.6%), satirical and parodic contents appear to be used more frequently to convey agency and stereotypical representations of female politicians (80% of satirical or parodic contents are related to the agentic dimension, see Figure 7).

**Figure 8:**
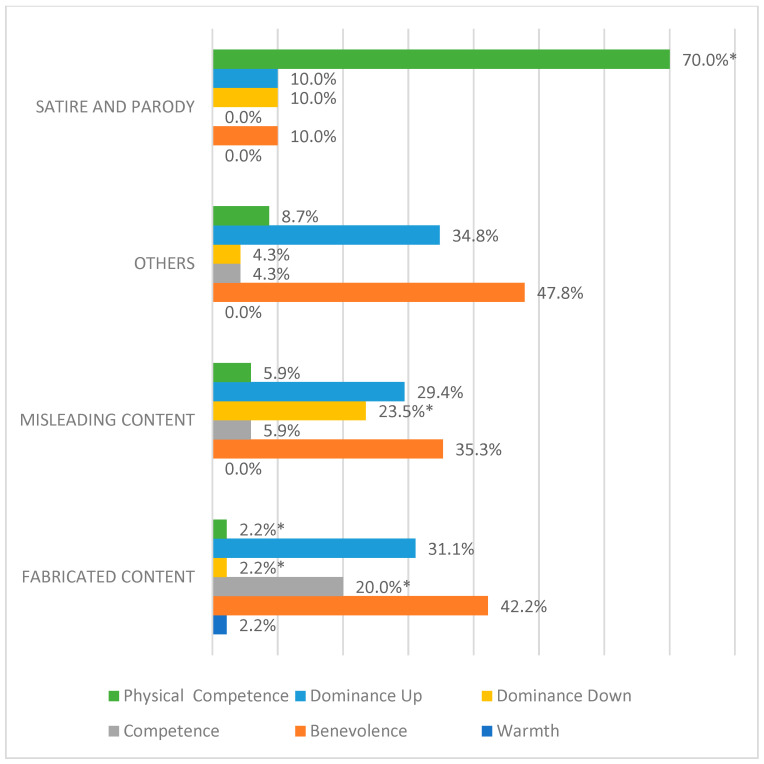
Percentage distribution of stereotypical sub-dimensions across different types of information disorder. * significant residuals at |2|.

In line with the previous results, the types of information disorders are significantly associated with the stereotype sub-dimensions (excluding no stereotyping contents), as revealed by both the chi-square test [χ^2^ (15) = 58.75; *p* < 0.000] and maximum likelihood ratio chi-square test [χ^2^ (15) = 43.88; *p* < 0.000]. The percentages highlight that fabricated news, i.e., those with a higher degree of falsification, have Benevolence (42.2%), then Dominance up (31.1%) and finally Competence (20%) as their main stereotype content. Concerning misleading news, the primacy of Benevolence (35.3%) and Dominance up (29.4%) is confirmed, but in this case, stereotype content associated with a lack of power (Dominance down, 23.5%) and Physical competence (5.9%) emerges too. Thus, these stereotype sub-dimensions appear to be more related to verisimilitude news, which is aimed at attributing evaluative labels rather than producing false truths. Stereotypical contents related to Physical competence are recurrently found to be associated with satire (see Figure 8); the role of this type of content should be further investigated, since it could trigger stereotyping processes associated with positive emotions, by significantly passing moral monitoring (D’Errico & Poggi, 2016; Matthes & Rauchfleisch, 2013).

## 4. Discussion

The recent literature has already shown how information disorders can play a role in reinforcing racist stereotypical representations and anti-immigrant attitudes (D’Errico et al., 2022; C. L. Wright & Duong, 2021), and, as highlighted by specific case studies, this also seems true in relation to sexist stereotypical representations, even within the political domain (Herrero-Diz et al., 2020; Stabile et al., 2019).

The present study aimed to investigate the role of information disorders in reflecting and reinforcing sexist stereotypical representations in the political field, by collecting and coding information disorders targeting Italian female politicians, using the two main dimensions emerging from the Stereotypes Content Model and Dual Perspective Model (Abele & Wojciszke, 2014; Fiske et al., 2007) and their recent application in social media linguistic data (Bosco et al., 2023), thus trying to fill the gap in the previous literature, mainly based on case studies (Stabile et al., 2019).

A preliminary qualitative analysis of the corpus’ lexicon revealed the prevalence of words related to the man-osphere’s dimensions, with a focus on female politicians’ looks, status, and money (Van Valkenburgh, 2021), with a great use of words that are related to the emotive, sexual, and familiar side, especially where the news pertains to the political issues.

Furthermore, words related to the dominance dimension are usually stressed, either by exaggerating or underestimating it, passing from the image of a ‘fascist/nazist’ who ‘force[s]’ someone to do something to a ‘butler who obeys’ to someone else.

These results anticipate what emerged in the quantitative content analysis: from a descriptive point of view, the main stereotypical sub-dimension emerging was Benevolence, followed by Dominance Up, Competence, and Physical Competence, thus confirming the tendency to distort, by excess and defect, the women power management. This aspect is expressed by stressing female politicians’ threatening power that can “take money from the poor and workers” and enforce decisions on people (e.g., “Meloni: ‘Starting today, it is required in all cars.’ Motorists are outraged, calling the imposition very expensive.”). The physical topic is conveyed by focusing on esthetics, using words related to leaders’ outfits or beauty symbols.

The prevalence of Benevolence and Dominance up contents suggests that the main stereotypical representation of female politicians concerns communion, thus representing them as dishonest, immoral, betraying Italians interests in favor of out-group members, despotic and, eventually, dangerous and overbearing. This stereotypical representation is in line with the aforementioned “Lady Macbeth narrative”, that villainizes power-seeking women, portrayed as callous, immoral and outrageous. In line with the double bind and backlash effect (Heldman et al., 2018; Jamieson, 1995; Rudman & Glick, 1999), female politicians who dare to act counter-stereotypically by engaging in traditionally male fields (the leadership and political ones), are punished by being represented as less communal, dangerous, and therefore worthy of people’s (and voters) anger and hostility.

Focusing on the news topic, it is also relevant that around 20% of information disorders concerned the esthetic or private life areas of female politicians; this finding is in line with the literature about gender differences in political media coverage that highlights how female politicians receive more appearance and family coverage than their male counterparts, thus shifting the focus from more relevant political topics, which instead are fundamental to enhance the female candidate’s credibility and competence (Dunaway et al., 2013; Lawrence, 2000; Van der Pas & Aaldering, 2020; Woodall & Fridkin, 2007). Information disorders seem to replicate the gendered topics pattern found, in general, media contents, potentially reinforcing the stereotypical association between women and the private sphere instead of public and political life (Van der Pas & Aaldering, 2020).

In addition to the description of stereotypical representations conveyed by information disorders targeting female politicians, the present study aimed to explore the potential role of leaders’ political orientation and type of information disorders. Results highlighted that the leader’s political orientation (conservative vs. progressive), despite not being significantly associated with the news topic and type of information disorders, seemed to play an important role in shaping the stereotype content related to female politicians. In particular, even though Benevolence and Dominance Up stereotype sub-dimensions appear to be prevalent mechanisms in female politicians regardless of their political orientation, progressive politicians appear to be more stereotyped in terms of Competence and Physical Competence; subsequently, even though Communion stereotype content is prevalent both in conservatives and progressives, Agency stereotype content appear to be prevalent in information disorders targeting progressive rather than conservative female politicians, particularly stressing the politician’s esthetic dimension (“*Stereotyping from my eyes*” effect). This difference in politicians’ stereotypical representations could be due to the different audience for which information disorders have been created: according to the motivated reasoning theory (Kahan, 2013), people prefer to be exposed and believe in contents that align their pre-existing beliefs and ideas; thus, information disorder targeting progressive leaders will probably address and suit a conservative audience, and vice versa. However, conservative and progressive voters seem to prioritize different traits in optimal leaders: Roets and Van Hiel (2009) find that left-wing voters in Belgium prefer politicians who are more friendly and open than right-wing voters. They also discovered that right-wing voters chose politicians with greater levels of machiavellianism (related with poorer agreeableness and honesty) and success drive (vigorous and dominant conduct). Laustsen’s (2017) study broadly supports these findings, demonstrating that conservatives place a higher value on candidate power and strong leadership (agency), whereas liberals (i.e., progressive voters) place a higher value on the leader’s warmth (communion). Therefore, it is reasonable to find a “*Stereotyping from my eyes*” effect, whereby a conservative audience, which favors agentic leaders, will stereotype progressive female politicians (their opponents) by depicting them as incompetent and less agentic, whereas a progressive audience, which places a higher value on leaders’ communion, will primarily stereotype conservative female politicians in terms of communion, describing them as immoral and callous. However, the “*Stereotyping from my eyes*” effect could also be due to a difference between conservative and progressive audience in adhering to traditional gender roles, with a conservative audience that embrace a more traditional way of viewing women (Lye & Waldron, 1997), thus potentially supporting a stereotypical representation of (progressive) women as less agentic than men and then less suitable for political roles (Eagly & Karau, 2002). A progressive audience, instead, could be more prone to recognize female politicians’ agency, being more likely to stereotype (conservative) female politicians in terms of communion (in line with the double bind and backlash effect; Jamieson, 1995; Rudman & Glick, 1999).

Finally, results highlight that information disorder’s types are significantly associated both to news topics and stereotypical representations: in particular, fabricated content and misleading content seemed to be associated with the professional and private life area, mostly conveying communion stereotype contents.

In particular, while in misleading news, where an evaluative manipulation of language is typical, a strong stereotyping of dominance emerges, both due to a lack and an excessive use of power; in the case of fabricated news, we found on the other side a high percentage of false news aimed at devaluing the politician’s competence. Thus, representations regarding female politicians’ lack of competence are conveyed mostly through ad hoc and completely false contents, created to propagate incorrect information: as aforementioned, this type of content is not limited by reality or objectivity, so it can be excessively harsh in its portrayal of political opponents, and this aspect is particularly useful to question politicians’ competence and skills (Acerbi, 2019; Osmundsen et al., 2021). On the other hand, dominance perception and evaluation (positive when the dominance levels are perceived as appropriate vs. negative when dominance levels are perceived as poor or excessive) seem to be more of a narrative frame matter: in fact, dominance-related representations are conveyed particularly through misleading contents, in which accurate information is linguistically framed to inaccurately represent an issue or an individual. Thus, the extent to which a behavior is perceived as properly or inappropriately dominant seems to depend on how the behavior is narrated and framed, as we can see in headlines like ‘*Disturbing statement: “We’ll re-educate your children.”’* (The original quote, stated by Monica Ciccirinnà, says “Be careful, Bussetti; if you truly want to safeguard women, you must enable sex education and gender education... If you were unfortunate enough to be born into one of those obscurantist families, public school must help you”). Satire and parodic contents, instead, were associated with the esthetic area, conveying agency stereotype content targeting female politicians, with a particular focus on politicians’ Physical competence (e.g., defining Elly Schlein as a “beautiful man” or comparing her to the Italian showman “Pippo Franco”). Thus, the role of Satire and parodic contents in spreading sexist stereotypes in the political field should be further investigated, considering how these types of content emphasize politicians’ appearance (that could be linked to the phenomenon of women dehumanization and sexual objectification), while triggering positive emotions in the audience, thus potentially bypassing the individual moral monitoring (D’Errico & Poggi, 2016; Matthes & Rauchfleisch, 2013).

## 5. Conclusions

The present study, although underlining the importance of investigating information disorders’ sexist stereotypical representations, presents some limitations. However, it is important to stress that the results obtained are preliminary, and any generalization to other contexts should be performed with caution.

Since there are no platforms or debunking websites that include all types of information disorders, collecting a complete and wide database is difficult and the sampling procedure for corpus content could be biased. In addition, the lack of a similar corpus targeting male politicians makes comparison difficult. Furthermore, the politicians’ personality can be a confounding factor in relation to the stereotypical representations targeting them. Future studies can collect a wider corpus, allowing a cross-cultural and cross-gender comparison, considering that a comparable sample of information disorders targeting Italian male politicians can be useful to highlight potential differences in stereotypical portrayals of women and men politicians. Despite this, the study sheds light on the role of information disorders in spreading sexist stereotypical content and thus potentially reinforcing gender disparities in politics, emphasizing the importance of preventing information disorders from being created and shared, as well as intervening toward users to recognize this type of content and the potentially stereotypical message that it conveys, as emerges from studies relating to other types of information disorders (D’Errico et al., 2024; Neylan et al., 2023). Furthermore, the “Stereotyping from my eyes” effect suggests that intervention aimed at countering sexist stereotypical representations in the political field should take into account users and leaders’ political orientations, offering different strategies to counter different types of stereotypical portraits. Future research can investigate whether this difference is also present in informative disorders targeting male politicians. Finally, future studies can deepen the potential roles of Artificial Intelligence, especially image generators, in disseminating manipulated and fabricated contents, reinforcing stereotypical and sexually objectifying representations.

## Data Availability

The authors are willing to share their data, analytics methods, and study materials with other researchers. The data supporting this study’s findings are available from the corresponding author, C.S. upon request.
